# Sex-specific outcomes in acute myocardial infarction-associated cardiogenic shock treated with and without V-A ECMO: a retrospective German nationwide analysis from 2014 to 2018

**DOI:** 10.1007/s00380-024-02509-z

**Published:** 2024-12-14

**Authors:** Hendrik Willem Beckmeyer, Jannik Feld, Jeanette Köppe, Andreas Faldum, Patrik Dröge, Thomas Ruhnke, Christian Günster, Holger Reinecke, Jan-Sören Padberg

**Affiliations:** 1 Department for Cardiology I: Coronary and Peripheral Vascular Disease, Heart Failure, University Hospital Münster, Albert-Schweitzer-Campus 1, Gebäude A1, 48149 Münster, Germany; 2 Institute of Biostatistics and Clinical Research, University of Münster, Schmeddingstraße 56, 49149 Münster, Germany; 3 AOK Research Institute (WIdO), AOK-Bundesverband, Rosenthaler Straße 31, 10178 Berlin, Germany

**Keywords:** V-A ECMO, Cardiogenic shock, Acute myocardial infarction, Sex, Outcome, Survival

## Abstract

Acute myocardial infarction-associated cardiogenic shock (AMICS) remains a condition with high mortality. Some patients require mechanical circulatory support (MCS) as their condition deteriorates. Sex-specific differences in risk factors and outcomes of cardiovascular disease have previously been described but are inconclusive regarding the use of MCS in AMICS. We aimed to investigate these with a focus on long-term outcomes. Health claim data from AOK – Die Gesundheitskasse (local health care funds) for patients hospitalized with AMICS between January 1, 2014, and December 31, 2015, was descriptively analyzed. Then, a Cox proportional hazards model was used to adjust for confounders. We analyzed 10,023 patients, of which 477 (4.8%) were treated with veno-arterial extra-corporeal membrane oxygenation (V-A ECMO). In-hospital mortality was high, but similar between treatments (V-A ECMO 59.1%, no V-A ECMO 56.6%). Women had a higher median age (78.9 years, IQR 13.8 vs. 71.8 years, IQR 17.9; *p* < 0.001), a different cardiovascular risk profile and in the conservatively treated patients underwent revascularization less often (69.2% vs. 77.1%; *p* < 0.001) than men did. In a multivariate analysis, female sex was not associated with lower survival (HR 1.03, CI 0.98–1.09; *p* = 0.233). V-A ECMO, however, was associated with lower survival in both sexes. We observed a low overall survival in follow-up after three years (no V-A ECMO: men 28.9% vs. women 21.7%, V-A ECMO: men 18.2% vs. women 17.0%). In conclusion, women with AMICS presented with a different risk profile, especially a higher age, and underwent guideline-recommended therapies such as revascularization less often than men. Female sex, however, was not associated with lower survival in a multivariate analysis. In-hospital mortality was high, regardless of treatment, and V-A ECMO was associated with lower survival in follow-up.

**Supplementary Information:**

The online version contains supplementary material available at 10.1007/s00380-024-02509-z.

## Introduction

Cardiogenic shock (CS) is a rare, but often fatal complication of acute myocardial infarction (AMI) and a major cause of death in patients with AMI. Its high incidence has remained relatively stable over the past decades [1]. Mortality, however, although decreasing recently, remains also high at approximately 40–50% [2].

Primarily, treatment of acute myocardial infarction-associated cardiogenic shock (AMICS) relies on coronary reperfusion. Early revascularization is critical, as it is the only intervention proven to significantly improve overall survival [3]. Pharmacological interventions are aimed at preserving or restoring organ function as well as perfusion through inotropes and/or vasopressors.

Despite optimal treatment, many patients remain in cardiogenic shock. In selected patients, temporary mechanical circulatory support (MCS), such as intra-aortic balloon pump (IABP), percutaneous left ventricular assist devices (pLVADs, e.g., Impella^®^, Abiomed, Inc., Danvers, MA, USA), or veno-arterial extracorporeal membrane oxygenation (V-A ECMO), is a viable option to maintain perfusion and temporarily replace or augment the failing heart’s function. The use of MCS is recommended to be considered depending on a patient’s age, comorbidities, prior neurological status, life expectancy and/or quality of life and is not considered the standard of care for all patients [4].

For the purposes of this analysis, we focused on V-A ECMO. It increases peripheral, splanchnic, coronary, and cerebral perfusion, it also augments oxygenation and supports the right heart thus providing the highest level of support and is applicable for use in the most critically ill patients, such as those with biventricular failure or rapidly deteriorating CS [5]. It, however, carries a high risk of complications, including LV-distention, vascular complications or hemolysis [6]. Since the decline in usage of IABP, V-A ECMO has been used in AMICS more often [7]. A recent meta-analysis described a lower mortality compared to IABP in CS [8]. However, three current randomized controlled trials have failed to show a survival benefit of V-A ECMO [9–11].

Female sex has been shown to be a risk factor for mortality in AMI [12], for less adherence to guideline-directed treatments [13] as well as for the development of AMICS and other complications [14]. There are conflicting data on the effect of sex on outcome in AMICS, however. Some authors report a higher mortality in women [15], while others report no difference in mortality [16]. It remains a matter of debate whether different treatments between sexes, as well as lower use of MCS in deteriorating AMICS [17] may contribute to worse outcomes.

The aim of this study was to investigate these possible differences in a representative sample of patients with AMICS treated with and without V-A ECMO in Germany. Thus, we analyzed health claim data from the AOK – Die Gesundheitskasse (local health care funds) for hospitalizations in the years 2014 and 2015 with follow-up until 2018.

## Methods

In the German health care system, hospitals are required to encode and submit to health insurers a principal diagnosis, which must be the diagnosis that led to hospital admission, based on a German version of the International Classification of Diseases, 10th Revision (ICD-10-GM), for reimbursement purposes. Additionally, any secondary diagnoses reflecting comorbidities or complications during hospitalization as well as any applicable diagnostic, interventional, or surgical procedures as listed in the Operational and Procedures Codes (*Operationen- und Prozedurenschlüssel*, OPS) are also encoded and then combined with other patient-specific data (such as age, sex, etc.) into a diagnosis-related group (G-DRG) according to which reimbursement is assigned. Therefore, complete reporting of data is ensured. Over-reporting is addressed by regular auditing of the submitted data.

Implantation of V-A ECMO and other, more complex procedures are remunerated separately to these calculated flat rates but are encoded and submitted similarly.

### Data source and patient selection

The AOK – Die Gesundheitskasse, a collective of regional health insurance companies, provide health insurance for approximately 27 million people [18], or almost one-third of the German population.

For this retrospective study, we obtained aggregated and anonymized data from the AOK Research Institute (WIdO) with cardiovascular diseases and selected all hospitalized patients aged 18 years or older, for which a combination of principal and secondary diagnoses of cardiogenic shock (ICD-10-GM code R57.0) and acute myocardial infarction (ICD-10-GM code I21.- or I22.-) was encoded between January 1, 2014, and December 31, 2015 (index-hospitalization).

Additionally, the use of V-A ECMO (OPS-code 8–852.3) was used as a parameter to form four groups for sex-specific comparative analysis: women and men, with and without V-A ECMO, respectively.

Baseline characteristics included a wide range of manifest cardiovascular disease and other relevant comorbidities and cardiovascular risk factors, obtained from ICD-codes submitted in the two years preceding index-hospitalization. For the same period, all relevant previous procedures relating to the principal diagnosis were registered. All applicable ICD-10-GM and OPS codes as well as their definitions are listed in Supplemental Table 1.

### In-hospital treatment and outcome

All OPS-encoded procedures during hospitalization and ICD-codes for stroke, bleeding, sepsis, and acute kidney injury, as well as interventions such as renal replacement therapy (RRT) or resuscitation, bleeding and blood transfusion were considered in-hospital treatment and outcome.

For revascularization, rates of percutaneous coronary intervention (PCI) and coronary artery bypass graft (CABG) within index-hospitalization, as well as early PCI, defined as PCI performed on the day of admission, were examined. All encoded procedures could only be specific to the exact day.

### Overall survival and follow-up

Mortality, primarily defined as index-hospitalization mortality, as well as the length of hospital stay were analyzed. For analysis of overall survival, patients were followed up from the date of admission of the index-hospitalization until the end of follow-up (December 31, 2018, exit from database, or death).

### Statistical methods

Qualitative data are presented as percentages and were tested via two-sided Chi-squared test. Quantitative data are presented as median + interquartile range (IQR) and were tested using a two-sided Wilcoxon test. A *p* value < 0.05 was considered statistically noticeable.

To protect patient anonymity, any subgroup representing ten or less patients was censored.

Overall survival rates were estimated with Kaplan–Meier estimators for selected time points (30 days, 1 year, 2 years, 3 years). Overall survival was then analyzed using multivariable Cox regression models, both for the complete study group, as well as the V-A ECMO-subgroup. The models included risk profiles of patients at baseline. All presented 95% confidence intervals (CI) and *p* values are standard unadjusted and purely descriptive. Hazard ratios (HRs) and unadjusted 95%-CI for all features are shown in the tables and figures.

All analyses were intended to be fully explorative.

Statistical analyses were performed using R version 4.0.2 (2020-06-22), R Foundation, Vienna, Austria, and Microsoft Excel for Mac (Version 16.73), Microsoft Corporation, Redmond, WA, USA.

## Results

### Baseline characteristics

A total of 10,023 patients with AMICS were analyzed, 477 (4.8%) of which were treated with V-A ECMO. Overall, 3804 (38.0%) of patients were female, whereas female representation was noticeably lower in patients treated with V-A ECMO (no V-A ECMO 38.6%, V-A ECMO 25.8%; *p* < 0.001). Altogether, 6481 (64.7%) patients had a primary diagnosis of ST-elevation myocardial infarction (STEMI), with no statistically apparent differences between sexes or treatment groups. In total, 8251 (82.3%) patients had preexisting CAD, and 7299 (72.8%) had CHF. Classic cardiovascular risk factors were also highly prevalent.

The complete baseline characteristics of our study group are shown in Table 1.

**Table 1:** Baseline characteristics

*N* = 10,023	No V-A ECMO			V-A ECMO		
	Men (*n* = 5865)	Women (*n* = 3681)	*p* value	Men (*n* = 354)	Women (*n* = 123)	*p* value
Age, years	72.3 (17.9)	79.2 (13.2)	< 0.001	63.7 (18.1)	67.0 (18.6)	0.046
Previous CAD	5026 (85.7%)	2779 (75.5%)	< 0.001	333 (94.1%)	113 (91.9%)	0.394
- 1- vessel	701 (12.0%)	525 (14.3%)	0.001	26 (7.3%)	14 (11.4%)	0.164
- 2-vessel	1083 (18.5%)	693 (18.8%)	0.659	56 (15.8%)	22 (17.9%)	0.593
- 3-vessel	3242 (55.3%)	1561 (42.4%)	< 0.001	251 (70.9%)	77 (62.6%)	0.087
Previous MI	1813 (30.9%)	1044 (28.4%)	0.008	111 (31.4%)	42 (34.1%)	0.568
Previous PCI	481 (8.2%)	236 (6.4%)	0.001	35 (9.9%)	17 (13.8%)	0.228
Previous CABG	599 (10.2%)	188 (5.1%)	< 0.001	18 (5.1%)	-	-
Hypertension	5040 (85.9%)	3379 (91.8%)	< 0.001	277 (78.2%)	109 (88.6%)	0.012
Smoking	1575 (26.9%)	508 (13.8%)	< 0.001	119 (33.6%)	39 (31.7%)	0.698
Diabetes mellitus	2898 (49.4%)	2066 (56.1%)	< 0.001	159 (44.9%)	67 (54.5%)	0.067
Obesity	1503 (25.6%)	1115 (30.3%)	< 0.001	95 (26.8%)	48 (39.0%)	0.011
Dyslipidemia	4002 (68.2%)	2499 (67.9%)	0.724	223 (63.0%)	85 (69.1%)	0.222
CHF	4181 (71.3%)	2724 (74.0%)	0.004	297 (83.9%)	97 (78.9%)	0.204
Atrial fibrillation/flutter	2038 (34.7%)	1387 (37.7%)	0.004	131 (37.0%)	40 (32.5%)	0.371
CKD	2373 (40.5%)	1755 (47.7%)	< 0.001	130 (36.7%)	35 (28.5%)	0.097
Previous Stroke	861 (14.7%)	602 (16.4%)	0.027	31 (8.8%)	11 (8.9%)	0.950
PAD I-II	731 (12.5%)	308 (8.4%)	< 0.001	33 (9.3%)	12 (9.8%)	0.887
PAD III-IV	486 (8.3%)	198 (5.4%)	< 0.001	26 (7.3%)	13 (10.6%)	0.261
History of cancer	1200 (20.5%)	700 (19.0%)	0.085	43 (12.1%)	22 (17.9%)	0.110

Data are presented as number (% of subgroup or median (IQR))

*CABG* coronary artery bypass graft, *CAD* coronary artery disease, *CHF* chronic heart failure, *CKD* chronic kidney disease; *IQR* interquartile range, *MI* myocardial infarction, *PAD* peripheral arterial disease, *PCI* percutaneous coronary intervention, *V-A ECMO* veno-arterial extracorporeal membrane oxygenation

- = censored

In the conservatively treated patients, women were noticeably older than men were (median ages: women 79.2 years, IQR 13.2; men 72.3 years, IQR 17.8; *p* < 0.001). The burden of atherosclerotic cardiovascular disease in men was greater, with higher rates of pre-existing CAD (85.7% vs. 75.5%; *p* < 0.001), especially 3-vessel-disease (55.3% vs. 28.4%; *p* < 0.001), a higher percentage of previous MI (30,9% vs. 28.4%; *p* = 0.008), and peripheral artery disease (PAD) of any stage (all *p* < 0.001). Smoking was about twice as prevalent in men (26.9% vs. 13.8%; *p* < 0.001). Women, in contrast, had higher rates of Diabetes mellitus and obesity (both *p* < 0.001). While CHF was, as described, highly prevalent in both sexes, it was more common in women (men 71.3%, women 74.0%; *p* = 0.004). Similarly, this was found for chronic kidney disease (CKD), and previous stroke.

The median age of patients treated with V-A ECMO was all in all lower than that of patients not treated with V-A ECMO (64.3 years, IQR 18.5 vs 75.2 years, IQR 17.5; *p* < 0.001). Women were, again, noticeably older than men (men 63.7 years, IQR 18.1; women 67.0 years, IQR 18.6; *p* = 0.046). Almost all patients in this group presented with known CAD, which, in both sexes, was considerably more often 3-vessel-disease than in the no V-A ECMO group (men 70.9%, women 62.6%; *p* < 0.001 compared to the no V-A ECMO group). CHF was more, while CKD was less prevalent than in patients not treated with V-A ECMO among both sexes. Fewer patients with known malignancies were treated with V-A ECMO.

### In-hospital treatment and outcome

Overall, 7528 (75.1%) patients underwent revascularization (either PCI or CABG) during index-hospitalization. A total of 6909 patients received PCI (68.9% total, 91.8% of revascularized patients) of which 6748 (97.7%) underwent PCI on the day of admission. 896 patients (8.9% total, 11.9% of revascularized patients) had CABG surgery; 278 patients (2.8% total, 3.7% of revascularized patients) received both PCI and CABG within the index-hospitalization.

Overall, hospitalization lasted a median of 8 days (IQR 19 days). Patients treated with V-A ECMO had a noticeably longer hospital stay (women with V-A ECMO 11 (IQR 29) days vs. women without V-A ECMO 5 (IQR 15) days, men with V-A ECMO 15.5 (IQR 33) days vs. men without V-A ECMO 9 (IQR 19) days; *p* < 0.001). Women across both groups had a shorter length of stay (women 5 (IQR 15) days vs. men 9 (IQR 20) days; *p* < 0.001).

Fewer women underwent revascularization in the no V-A ECMO group (69.2% vs. 77.1%; *p* < 0.001), with lower rates of both PCI (overall and on the day of admission) and CABG (all *p* < 0.001). IABP use likewise was less common in women in this group (6.6% vs. 9.2%; *p* < 0.001).

In the V-A ECMO group there was a higher rate of revascularization of any kind (95.8% vs. 74.1% in the no V-A ECMO group; *p* < 0.001). Particularly CABG was markedly more common in this group (V-A ECMO 35.4% vs. no V-A ECMO 7.6%; *p* < 0.001). No difference between women and men in rates of revascularization could be found in this group.

Overall, complications were more common in the V-A ECMO group. These included bleeding, resulting in almost all patients, but women more than men (95.9% vs. 89.0%; *p* = 0.02), receiving packed red blood cell (PRBC) transfusion in this group, as well as resuscitation (64.2% vs. 58.8%; *p* < 0.001 compared to the no V-A ECMO group). Sepsis and stroke, similarly, were encoded more often in this group, and so were acute kidney injury (AKI) and renal replacement therapy (RRT) (all *p* < 0.001 compared to the no V-A ECMO group). Except for the observed rate of PRBC transfusion, no differences in complications between sexes manifested. The complete subgroup analyses can be found in Table 2.

**Table 2:** In-hospital treatment and outcome

*N* = 10,023	No V-A ECMO			V-A ECMO		
	Men (*n* = 5865)	Women (*n* = 3681)	*p* value	Men (*n* = 354)	Women (*n* = 123)	*p* value
STEMI	3757 (64.1%)	2399 (65.2%)	0.268	244 (68.9%)	81 (65.9%)	0.529
NSTEMI	2108 (35.9%)	1282 (34.8%)	0.268	110 (31.1%)	42 (34.1%)	0.529
Revascularization	4524 (77.1%)	2547 (69.2%)	< 0.001	336 (94.9%)	121 (98.4%)	0.099
PCI	4151 (70.8%)	2403 (65.3%)	< 0.001	259 (73.2%)	96 (78.0%)	0.285
PCI on day of admission	4069 (69.4%)	2347 (63.8%)	< 0.001	244 (68.9%)	88 (71.5%)	0.587
CABG	515 (8.8%)	212 (5.8%)	< 0.001	124 (35.0%)	45 (36.6%)	0.756
PCI and CABG	142 (2.4%)	68 (1.8%)	0.063	47 (13.3%)	21 (17.1%)	0.300
IABP	539 (9.2%)	242 (6.6%)	< 0.001	91 (25.7%)	35 (28.5%)	0.551
AKI	1744 (29.7%)	1005 (27.3%)	0.011	195 (55.1%)	70 (56.9%)	0.726
RRT	896 (15.3%)	413 (11.2%)	< 0.001	202 (57.1%)	60 (48.8%)	0.112
Stroke	271 (4.6%)	164 (4.5%)	0.706	45 (12.7%)	15 (12.2%)	0.882
In-hospital resuscitation	2526 (43.1%)	1478 (40.2%)	0.005	208 (58.8%)	79 (64.2%)	0.286
Bleeding	548 (9.3%)	426 (11.6%)	< 0.001	143 (40.4%)	57 (46.3%)	0.250
PRBC transfusion	1417 (24.2%)	908 (24.7%)	0.574	315 (89.0%)	118 (95.9%)	0.022
Sepsis	670 (11.4%)	320 (8.7%)	< 0.001	94 (26.3%)	26 (21.1%)	0.233
Death (discharge status index-hospitalization)	3113 (53.1%)	2286 (62.1%)	< 0.001	205 (57.9%)	77 (62.6%)	0.362
Death (discharge status index-hospitalization), both sexes in group	5399 / 9546 (56.56%)			282 / 477 (59.12%)		0.270

Data are presented as number (% of subgroup)

*AKI* acute kidney injury, *CABG* coronary artery bypass graft, *IABP* intra-aortal balloon pump, *IQR* interquartile range, *NSTEMI* non-ST-elevation myocardial infarction, *PCI* percutaneous coronary intervention, *PRBC* packed red blood cells, *RRT* renal replacement therapy, *STEMI* ST-elevation myocardial infarction, *V-A ECMO* veno-arterial extracorporeal membrane oxygenation

### In-hospital mortality and overall survival in follow-up

**Fig. 1:**
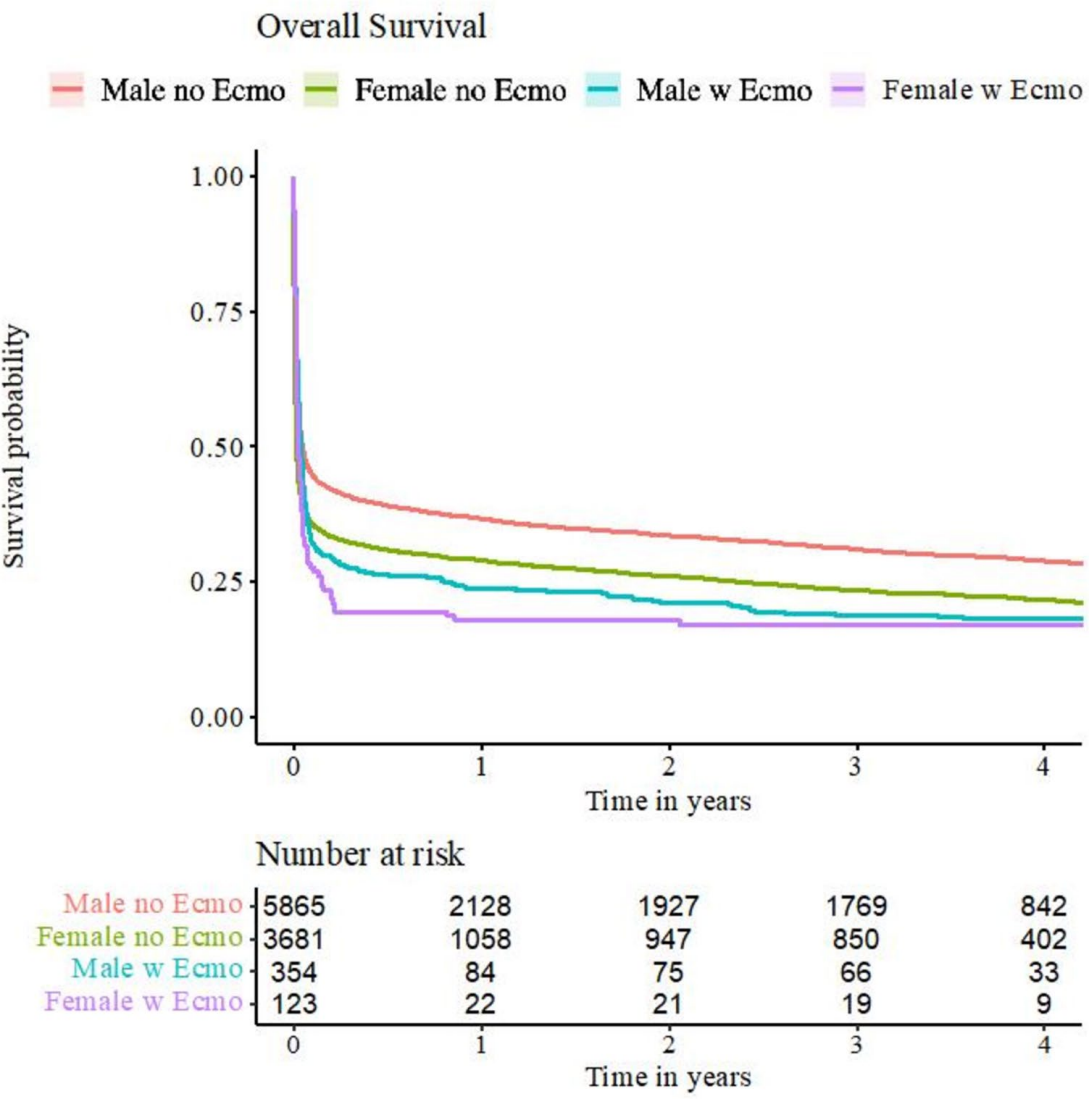
Kaplan–Meier model predicting overall survival for follow-up of 1–4 years. Men without ECMO-therapy displayed the highest estimated survival probability, women with ECMO-therapy the lowest. In patients without V-A ECMO, differences in survival between sexes persisted throughout follow-up, whereas this was not the case in patients with V-A ECMO

Overall mortality during index hospitalization was 56.7%. Kaplan Meier model estimators for overall survival are shown in Fig. 1, while point estimators as well as CIs are presented in Supplemental Table 2.

Between treatment groups, unadjusted in-hospital mortality descriptively did not differ (V-A ECMO 59.1%, no V-A ECMO 56.6%). A noticeably lower survival in patients treated with V-A ECMO, however, was observed in follow-up (30 days: no V-A ECMO 41.9% vs. V-A ECMO 32.5%; 1 year: 33.4% vs. 22.2%; 2 years: 30.1% vs. 20.1%; 3 years: 27.4% vs. 19.9%).

Analyzing the unadjusted data of the entire study population, women descriptively displayed higher in-hospital mortality (62.1% vs. 53.4%) while being in the median 6.9 years older than men. Additionally, this was only seen in women treated without V-A ECMO (62.1% vs. 53.1%) whereas it was not in women in the V-A ECMO group (62.6% vs. 57.9%).

Disregarding treatment modality, men descriptively displayed higher rates of overall survival throughout follow-up in an unadjusted analysis (30 days: 44.8% vs. 36.0%; 1 year: 35.6% vs. 28.4%; 2 years: 32.2% vs. 25.4%; 3 years: 29.5% vs. 22.8%).

However, a Cox proportional hazards model to adjust for confounders could not identify sex as a risk factor for mortality (HR 1.03, CI 0.98 – 1.09; *p* = 0.233).

Treatment with V-A ECMO was associated with lower survival in both sexes (HR 1.59, CI 1.41 – 1.79 in men, HR 1.51, CI 1.24 – 1.85 in women; *p* < 0.001).

For both sexes, higher age (HR 1.04, CI 1.04 – 1.04 in men, HR 1.04, CI 1.03 – 1.04 in women; *p* < 0.001), a history of diabetes mellitus (HR 1.22, CI 1.14 – 1.30 in men; HR 1.20, CI 1.11 – 1.30 in women; *p* < 0.001) or stroke (HR 1.16, CI 1.07 – 1.26 in men; HR 1.32, CI 1.20 – 1.45 in women; *p* < 0.001) could be identified as additional potential risk factors for overall survival.

Amongst others, previous MI (HR 0.76, CI 0.71 – 0.82 in men; HR 0.63, CI 0.58 – 0.69 in women; *p* < 0.001), and CHF (HR 0.68, CI 0.64 – 0.73 in men; HR 0.66, CI 0.61 – 0.72 in women; *p* < 0.001) could be identified as potentially protective factors for both sexes.

**Fig. 2:**
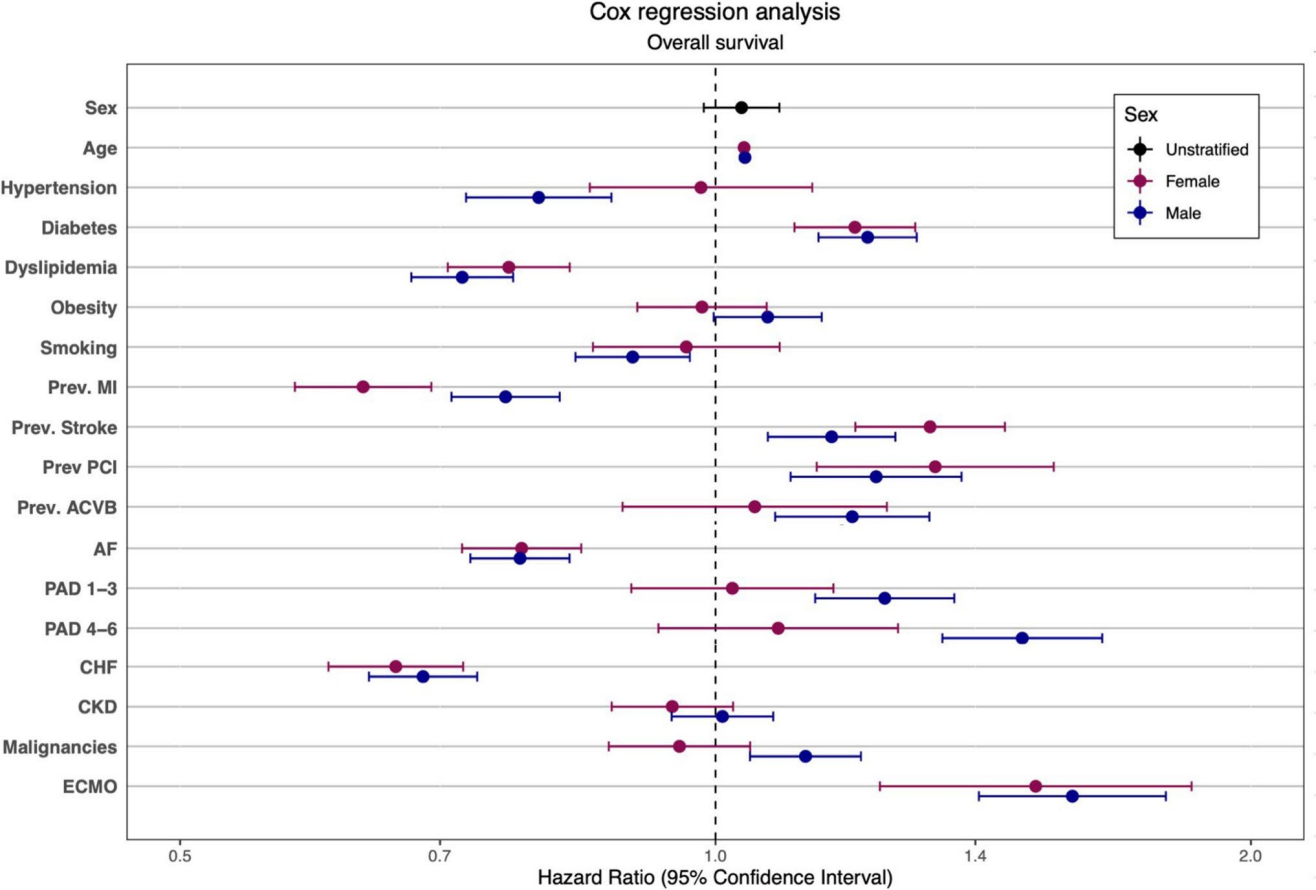
A Cox-regression for the entire study population identified several factors associated with lower survival, such as age, Diabetes, previous Stroke and PCI in both sexes. Treatment with V-A ECMO similarly was associated with lower survival in both sexes. Previous MI as well as AF, CHF and Dyslipidemia showed an association with increased survival in both sexes

The complete results of the Cox proportional hazards model are displayed in Fig. 2 and in Supplemental Table 3.

Within the V-A ECMO group only, a Cox proportional hazards model showed age to be associated with a higher risk of mortality for both sexes, as well (HR 1.02, CI 1.01 – 1.03 in men, *p* = 0.003; HR 1.02, CI 1.00 – 1.04 in women, *p* = 0.016). In men, diabetes was associated with a higher risk (HR 1.51, CI 1.18 – 1.95; *p* = 0.001), and CHF with a lower risk of mortality (HR 0.42, CI 0.31 – 0.58; *p* < 0.001). All other investigated parameters showed no associations in this group and no sex-specific differences could be found (Supplemental Table 4).

## Discussion

In this retrospective analysis of administrative data of 10,023 patients hospitalized for AMICS in Germany from 2014 to 2015 treated with and without V-A ECMO we observed several sex-related findings, both in baseline characteristics, as well as in outcomes. Women were older, had a different cardiovascular risk profile and, in the conservative group, lower rates of revascularization. However, after adjustment for comorbidities, no differences between both sexes regarding mortality could be observed. Additionally, unadjusted in-hospital mortality between treatment modalities was similar, but V-A ECMO was associated with lower survival in multivariate analysis as well as follow-up.

In our data, in the conservatively treated patients, women were clearly older than men were. However, after adjustment for age and further risk factors, female sex was not associated with a higher risk for death, though higher age was.

Additionally, we found diabetes mellitus and previous stroke to be associated with higher mortality. Both were documented more often in women, probably also due to higher age. This is in accordance with multiple previous analyses in CS, describing differing risk factors [19], including age [20], clinical presentation, angiographic findings and disease progression [21] in women. These possibly lead to longer treatment times, including longer time to revascularization, as well as lower rates of revascularization in general [22].

The previously mentioned was observed in our data, too. Women underwent revascularization of any kind less often than men did. When analyzing treatment groups separate from each other, however, this was observed in conservatively treated women, only. Though no direct association can be made in our data, it is conceivable that the above-mentioned older age and higher rates of diabetes mellitus, as well as obesity and CKD may have influenced this. Regardless of possible influences on physician’s treatment decisions, these, for example, may also lead to problems concerning vessel access. This under-treatment is in clear opposite to the finding that a similar, if not higher, benefit of early revascularization for women has been described [23].

Revascularization rates were markedly higher with no difference between sexes in patients treated with V-A ECMO. Consequently, no differences in mortality between sexes were observed in the V-A ECMO patients, confirming the positive effect of the deliverance of evidence-based care on mortality [13].

Additionally, V-A ECMO was used less often in women. As it could be shown to be associated with lower survival in our analysis, this emphasizes the impact a higher age and comorbidities had on mortality in women.

The underrepresentation of women in cardiovascular studies [24] is underlined by the results of our analysis of real-world remuneration data, serving as a representative sample of all hospitalizations due to AMICS within the investigated period. Using multivariable models to account for possible confounders, a sex-inherent risk for mortality in AMICS could not be identified. This needs to be considered in further studies to assure sex equality in recruitment.

### In-hospital mortality

Overall, in-hospital mortality in our cohort was 56.7%. Contemporary database analyses report a range of mortality rates in AMICS. An analysis of US data described a decline in mortality from 49 to 37% between 2004 and 2018 [25]. In German data from 2005 to 2017, a consistent in-hospital mortality rate of about 56–57% was described [26]. Comparison of this outcome metric between studies is difficult, however, due to an inhomogeneity of the disease entity of CS. Differing definitions may lead to the inclusion of different patient groups, and, furthermore, disease severity is a major factor. To stratify both, the Society for Coronary Angiography and Interventions (SCAI) published a consensus paper for a five-stage classification system [27] which has been validated in patients with AMICS [28] and correlates well with observed mortality. A higher proportion of patients with more severe CS might explain our reported high mortality rates. Indicative of this, for instance, we reported generally high rates of in-hospital resuscitation in both treatment groups. Additionally, many risk factors we could identify to be associated with lower survival were highly prevalent in our patient groups. These include, for example, a generally high patient age, as well as a high percentage of documented diabetes mellitus.

V-A ECMO was associated with lower survival in multivariate analysis. It needs to be considered that patients treated with V-A ECMO irrespective of sex were considerably younger, and had less frequently documented, for example, CKD and malignancies. This may be seen as a potential source of bias, leading to more aggressive treatment. We, for example, found higher rates revascularization and IABP-usage in this group. Furthermore, the rates of performed resuscitation were higher in this group, which may signify a more severe course of disease but on the other hand may also suggest a greater willingness by the treating physicians to perform this very aggressive treatment in this group. As the length of hospital stay was also noticeably longer in the V-A ECMO patients, physician’s decision to continue treatment for longer may have also played a role.

### Overall survival

Although we did not observe any differences in in-hospital mortality between treatment groups, these manifested during long-term follow-up, as V-A ECMO patients displayed higher descriptive rates of death. This compares to previously published data in which a significant post-hospitalization mortality in patients with AMICS was observed [29]. Our data confirm that patients with AMICS in general display high rates of post-hospitalization mortality, but also that patients treated with V-A ECMO fare worse, still.

Reasons for the observed low survival in follow-up possibly lie in the severity of CS in the described patients and a generally high patient age, but might be inherent to CS itself, no matter its severity. Briasoulis et al. reported high rates of post-CS heart failure and subsequent hospitalization, especially in women [30], which themselves incur high morbidity and mortality [31].

As to why V-A ECMO patients had a worse outcome might be inherent in the treatment modality itself, as previously stated. It appears any possible offset of the risk incurred by ECMO by, for example, a lower patient age or more aggressive treatment vanished in follow-up. A possible explanation for this is the large number of complications that V-A ECMO entails. Although we could not include and differentiate all potential and described complications of V-A ECMO, several outcome-relevant complications were much more frequent in the V-A ECMO patients. These included bleeding, the impact of which on mortality has previously been described [32], as well as sepsis. ECMO itself is a risk factor for nosocomial infections and sepsis, which in turn are associated with much lower in hospital survival [33] as well as survival in follow-up [34]. Stroke, as a surrogate parameter for thrombo-embolic events (though distal limb perfusion complications have a different etiology and could unfortunately not be analyzed here), and AKI were also more common in the V-A ECMO patients.

## Limitations

As stated, the most important limitation of our study is the fact that we analyzed retrospective remuneration data. Although the use of claims data and ICD-10-codes in particular [35], correlates well with a patient’s clinical picture, this nonetheless made stratifying the included patients according to current recommendations such as the SCAI classification impossible, thus impeding comparability between studies. This, however, has been a limitation of almost all studies.

Moreover, we could not analyze important complications such as those relating to extremity perfusion, as there is no clear representation of these in the ICD-10-codes. Data on angiographic findings were also not available, neither was detailed data on revascularization and/or inability to revascularize at all.

Also, since our data represents a synopsis of a patient’s hospitalization, the timing of different interventions could not be more accurate than to the exact day. Thus, we were not able to discriminate, for example, if PCI was performed within the recommended time after diagnosis, or why a significant proportion of patients received both PCI and CABG. This may have been due to a complex coronary anatomy, failed PCI, and CABG as “bail-out”, or, vice versa, an early occlusion of a CABG-vessel that needed to be treated with PCI, representing very different problems with impacts on prognosis.

The analysis of the use of other, additional MCS was impeded by this, as well. This concerns not only IABP, as its use in the V-A ECMO patients might signify a failed attempt to stabilize with IABP and subsequent escalation to V-A ECMO or implantation of IABP as LV-protection, i.e. LV-unloading, after V-A ECMO was already running. Since IABP was shown, however, to not differ from standard care regarding mortality [36] before the queried period, we decided to include it. Regarding Impella^®^, however, since we were not able to clearly distinguish its use in protected PCI and its use as MCS in CS, we were not able not include it in our query. This would have led to diluted treatment groups. As MCS usage since the recent publication of the ECLS-SHOCK [9] and DanGer Shock trials [37] possibly changed to favor Impella^®^, its analysis would have been interesting, nonetheless. On the other hand, the use of Impella^®^ in the queried period was still low, overall, as its use has only recently increased [7, 26].

## Conclusion

We present data from a large real-world cohort of patients with AMICS, who received either conservative treatment or V-A ECMO and analyzed these data regarding sex-specific differences in outcome.

Women presented with a different risk profile than men did and were treated differently, e.g. with lower rates of revascularization. Female sex, however, could not be identified as a risk factor after adjustment for confounders. Therefore, the observed differences in treatment are not justified and more attention should be given to balance any sex inequalities in treatment. Additionally, long-term survival was low in both treatment groups, but V-A ECMO was associated with even lower survival in follow-up, possibly due to the large number of complications incurred.

## Supplementary Information

Below is the link to the electronic supplementary material.

Supplementary file1 (PDF 281 KB)

## Data Availability

All data are stored in a central database at the AOK Research Institute (WIdO, Berlin). We received aggregated and anonymized data of all patients meeting the above-mentioned inclusion criteria. The authors confirm that the data utilized in this study cannot be made available in the manuscript, the supplemental files, or in a public repository due to German data protection laws (‘Bundesdatenschutzgesetz’, BDSG). Generally, access to data of statutory health insurance funds for research purposes is possible only under the conditions defined in German Social Law (SGB V § 287). Requests for data access can be sent as a formal proposal specifying the recipient and purpose of the data transfer to the appropriate data protection agency. Access to the data used in this study can only be provided to external parties under the conditions of the cooperation contract of this research project and after written approval by the sickness fund. For assistance in obtaining access to the data, please contact wido@wido.bv.aok.de.

## Abbreviations

AMI: Acute myocardial infarction
AMICS: Acute myocardial infarction-associated cardiogenic shock
AOK: AOK–Die Gesundheitskasse
CABG: Coronary artery bypass graft
CAD: Coronary artery disease
CHF: Chronic heart failure
CKD: Chronic kidney disease
CS: Cardiogenic shock
G-DRG: German Diagnosis Related Groups
ICD-10-GM: International Classification of Diseases, 10th Revision, German Modification
MCS: Mechanical circulatory support
MI: Myocardial infarction
OPS: Operationen- und Prozedurenschlüssel (*Operations and procedures codes*)
PAD: Peripheral artery disease
PCI: Percutaneous coronary intervention
pLVAD: Percutaneous left ventricular assist device
PRBC: Packed red blood cells
RRT: Renal replacement therapy
SCAI: Society for Coronary Angiography and Interventions
V-A ECMO: Veno-arterial extra-corporeal membrane oxygenation
WIdO: Wissenschaftliches Institut der AOK
