# Supplementary material for: Sex-specific outcomes in acute myocardial infarction-associated cardiogenic shock treated with and without V-A ECMO: a retrospective German nationwide analysis from 2014 to 2018
Source: Heart Vessels. 2024 Dec 14;40(7):559–69. doi: 10.1007/s00380-024-02509-z (PMC12166010; doi:10.1007/s00380-024-02509-z)
Supplement: Supplementary file 1 — Supplementary file1 (PDF 281 KB) [file 380_2024_2509_MOESM1_ESM.pdf]

Supplemental material – Heart and Vessels

Sex-specific outcomes in acute myocardial infarction-associated cardiogenic shock treated with and without V-A ECMO – a retrospective German nationwide analysis from 2014 to 2018.

Hendrik Willem Beckmeyer[a]; Jannik Feld, MSc[b]; Jeanette Köppe, PhD[b]; Andreas Faldum, PhD[b]; Patrik Dröge[c]; Thomas Ruhnke[c]; Christian Günster[c]; Holger Reinecke, MD[a]; Jan-Sören Padberg, MD[a]

[a] Department for Cardiology I: Coronary and Peripheral Vascular Disease, Heart Failure, University Hospital Münster, Albert-Schweitzer-Campus 1, Gebäude A1, 48149 Münster, Germany

[b] University of Münster, Institute of Biostatistics and Clinical Research, Schmeddingstraße 56, 49149 Münster, Germany

[c] AOK Research Institute (WIdO), AOK-Bundesverband, Rosenthaler Straße 31, 10178 Berlin, Germany

Address for correspondence:

Hendrik Willem Beckmeyer

Albert-Schweitzer-Campus 1

D-48149 Münster, Germany

Phone: +49 251 83 43244

E-Mail: hendrik.beckmeyer@ukmuenster.de

ORCID: 0000-0002-9754-7074

Table of figures

Supplemental Table 1: ICD-10-GM- and OPS-codes

Supplemental Table 2: Kaplan-Meier estimators of survival

Supplemental Table 3: Cox regression (overall)

Supplemental Table 4: Cox regression (ECMO-group)

Supplemental Table 1: Applicable ICD-10-GM- and OPS-Codes

Diagnosis	ICD-10-GM-2023-code(s)
Cardiogenic shock	R57.0
ST-elevation myocardial infarction	I21.0, I21.1, I21.2, I21.3
Non-ST-elevation myocardial infarction	I21.4
Coronary artery disease	I25.1-
- 1-vessel	I25.11
- 2-vessel	I25.12
- 3-vessel	I25.13
Previous myocardial Infarction	I21.-, I22.-
Hypertension	I10.-, I11.-, I12.-, I13.-, I14.-, I15.-
Smoking	F17.-
Diabetes mellitus	E10.-, E11.-, E12.-, E13.-, E14.-
Obesity	E66.-
Dyslipidemia	E78.-
Chronic heart failure	I50.-
Atrial flutter / fibrillation	I48.-
Chronic kidney disease	N18.-, N19.-
Previous Stroke	I69.3; I69.4
Peripheral artery disease, stage Fontaine I-II	I70.20-22
Peripheral artery disease, stage Fontaine III-IV	I70.23-25
History of malignancies	C0-C97
Acute kidney injury	N17.-
Acute stroke (hemorrhagic and ischemic)	I60.-, I61.-, I62.-, I63.-, I64.-
In-hospital resuscitation	I46.-
Bleeding	K92, H44.8, T81.0, T81.2, T81.3, T81.7
Sepsis	A41.-
**Procedure**	**OPS-code**
Percutaneous coronary intervention	8-837
Coronary artery bypass graft	5-36
V-A ECMO	8-852.3
Renal replacement therapy	8-853, 8-854, 8-855
PRBC transfusion	8-800

According to the International Classification of Diseases, 10th Revision, German Modification (ICD-10-GM) and the Operations and Procedure Code (OPS)

IABP = intra-aortal balloon pump; PRBC = packed red blood cells; V-A ECMO = veno-arterial extracorporeal membrane oxygenation

Supplemental Table 2: Kaplan-Meier estimators of survival

	No V-A ECMO		V-A ECMO	
	**Men (95%-CI)**	**Women (95%-CI)**	**Men (95%-CI)**	**Women (95%-CI)**
30 days	45.5% (44.2 – 46.7%)	36.3% (34.7 – 37.8%)	33.9% (29.0 – 38.8%)	28.5% (20.5 – 36.4%)
1 year	36.7% (35.5 – 37.9%)	29.0% (27.5 – 30.5%)	23.7% (19.3 – 28.2%)	17.9% (11.1 – 24.7%)
2 years	33.6% (32.3 – 34.8%)	26.1% (24.7 – 27.5%)	21.2% (16.9 – 25.4%)	17.9% (11.1 – 24.7%)
3 years	28.9% (27.7 – 30.1%)	21.7% (20.4 – 23.1%)	18.2% (14.1 – 22.2%)	17.0% (10.4 – 23.7%)

Kaplan-Meier estimators are given as % and 95%-CI.

Abbreviations: V-A ECMO = veno-arterial extracorporeal membrane oxygenation

Supplemental Table 3: Cox regression overall survival (all patients)

	Men			Women			*p* value (diff.)
	HR	95%-CI	*p* value	HR	95%-CI	*p* value	
Age	1.04	1.04 – 1.04	< 0.001	1.04	1.03 – 1.04	< 0.001	0.672
Hypertension	0.80	0.72 – 0.87	< 0.001	0.98	0.85 – 1.13	0.798	0.011
Diabetes mellitus	1.22	1.14 – 1.30	< 0.001	1.20	1.11 – 1.30	< 0.001	0.772
Dyslipidemia	0.72	0.67 – 0.77	< 0.001	0.77	0.71 – 0.83	< 0.001	0.248
Obesity	1.07	1.00 – 1.15	0.059	0.98	0.90 – 1.07	0.682	0.127
Smoking	0.90	0.83 – 0.97	0.005	0.96	0.85 – 1.09	0.540	0.331
Previous MI	0.76	0.71 – 0.82	< 0.001	0.63	0.58 – 0.69	< 0.001	0.001
Previous Stroke	1.16	1.07 – 1.26	< 0.001	1.32	1.20 – 1.45	< 0.001	0.052
Previous PCI	1.23	1.10 – 1.38	< 0.001	1.33	1.14 – 1.55	< 0.001	0.436
Previous CABG	1.19	1.08 – 1.32	< 0.001	1.05	0.89 – 1.25	0.561	0.233
Atrial fib./flutter	0.78	0.73 – 0.83	< 0.001	0.78	0.72 – 0.84	< 0.001	0.941
PAD 1-3	1.25	1.14 – 1.36	< 0.001	1.02	0.90 – 1.16	0.744	0.013
PAD 4-6	1.49	1.34 – 1.65	< 0.001	1.08	0.93 – 1.27	0.305	0.001
CHF	0.68	0.64 – 0.73	< 0.001	0.66	0.61 – 0.72	< 0.001	0.382
CKD	1.01	0.94 – 1.08	0.788	0.95	0.87 – 1.02	0.163	0.155
History of cancer	1.12	1.05 – 1.21	0.002	0.95	0.87 – 1.05	0.318	0.008
V-A ECMO	1.59	1.41 – 1.79	< 0.001	1.51	1.24 – 1.85	< 0.001	0.692
	HR			95%-CI			*p* value
Sex	1.03			0.98 – 1.09			0.233

Data are presented as HR and 95%-CI. Differences between sexes were tested with interaction terms.

Abbreviations: CABG = coronary artery bypass graft; CHF = chronic heart failure; CKD = chronic kidney disease; CVD = cerebrovascular disease; MI = myocardial infarction; PAD = peripheral arterial disease; PCI = percutaneous coronary intervention

Supplemental Table 4: Cox regression overall survival (V-A ECMO-group)

	**Men**			**Women**			***p* value (diff.)**
	**HR**	**95%-CI**	***p* value**	**HR**	**95%-CI**	***p* value**	
Age	1.02	1.01 – 1.03	0.003	1.02	1.00 – 1.04	0.016	0.581
Hypertension	0.75	0.55 – 1.01	0.058	0.90	0.47 – 1.73	0.749	0.614
Diabetes mellitus	1.51	1.18 – 1.95	0.001	1.35	0.89 – 2.06	0.161	0.653
Previous MI	0.82	0.61 – 1.10	0.181	0.73	0.42 – 1.27	0.260	0.703
Previous Stroke	0.99	0.66 – 1.48	0.955	0.87	0.42 – 1.80	0.705	0.761
Previous PCI	1.23	0.81 – 1.87	0.337	1.44	0.67 – 3.06	0.349	0.725
Previous CABG	1.58	0.94 – 2.63	0.082	0.46	0.11 – 2.00	0.303	0.122
CHF	0.42	0.31 – 0.58	< 0.001	0.65	0.40 – 1.08	0.098	0.152
CKD	0.82	0.64 – 1.06	0.134	0.73	0.46 – 1.16	0.184	0.652

Data are presented as HR and 95%-CI. Differences between sexes were tested with interaction terms.

Abbreviations: CABG = coronary artery bypass graft; CHF = chronic heart failure; CKD = chronic kidney disease; MI = myocardial infarction; PCI = percutaneous coronary intervention
